# Effects of PM2.5 on mucus hypersecretion in airway through miR-133b-5p/EGFR/Claudin1/MUC5AC axis

**DOI:** 10.18632/aging.205785

**Published:** 2024-05-26

**Authors:** Lerong Chen, Liangliang Wu, Xiaopeng Cheng, Jianhua Huang, Jianping Peng

**Affiliations:** 1 Department of Respiratory, Jiangxi Provincial Chest Hospital, Nanchang 330006, Jiangxi, China

**Keywords:** PM2.5, miR-133b-5p, EGFR, MUC5AC, airway inflammation

## Abstract

Objective: To investigate the role of the EGFR/MAPK signaling pathway in PM2.5 in promoting the MUC5AC hypersecretion in airway and exacerbating airway inflammation.

Methods: By establishing rat model exposed to PM2.5, overexpressing miR-133b-5p and Claudin1, the content of IL-1 and TNF-α in serum were detected by ELISA, the pathology of lung tissue was observed by HE staining, p-EGFR, Claudin1, MUC5AC, p-ERK1/2, p-JNK, p-p38 in rats lung tissue were detected by immunohistochemical and WB, the expression level of miR-133b-5p in rats lung tissue were detected by qPCR.

Results: After the rats were exposed to PM2.5, the content of inflammatory factors in serum increased, the inflammatory damage of lung tissues occurred, the expression of miR-133b-5p was down-regulated, and the expression of MUC5AC protein was increased. The ELISA test results showed that the expression of IL-1 and TNF-α in the model group was significantly higher than that in the control group, and the model +AG1478 treatment group was down-regulated compared with the model group, and the +miR-133b-5p agomir treatment group was significantly lower than that in the control group, the model group and the model +Claudin1 overexpression blank load group, and the model +Claudin1 overexpression group was down-regulated compared with the model group and the model +Claudin1 overexpression blank load group. The protein detection results showed that the expression of p-EGFR, MUC5AC, p-ERK1/2, p-JNK and p-p38 proteins was increased and the expression of Claudin1 protein was decreased in the model group compared with the control group. In the model + AG1478 treatment group, model + miR-133b-5p agomir treatment group and model + Claudin1 overexpression group, compared with the model group, p-EGFR, MUC5AC, p-ERK1/2, p-JNK, p-p38 protein expression was down-regulated, and Claudin1 protein expression was up-regulated.

Conclusions: PM2.5 inhibited the expression of miR-133b-5p to activate the EGFR/MAPK signal pathway, induce the hypersecretion of MUC5AC, thus aggravating PM2.5-related airway inflammation in rats.

## INTRODUCTION

As socioeconomic development and industrialization processes continue to advance, environmental pollution, especially problems of air pollution, have been threatening human beings. Fine particulate matter (PM2.5), with an aerodynamic diameter ≤ 2.5 μm, is one of the main components of air pollution. Due to its small size and easy transportation, PM2.5 can be inhaled deep into the human airway and deposited in the lung tissues, especially the alveolar regions, causing local damage to the lungs [1]. There is evidence that PM2.5 has harmful effects on lung function and alveolar structure, and the mechanisms involved focus on inflammatory responses, oxidative stress, immune disorders, and genetic alterations [2–5]. Our previous study found that airway inflammation also increased in healthy rats as the concentration of exposed PM2.5 increased [6].

As the front line of defense against inhaled pathogens and particulate matter, airway epithelial cells could trigger airway inflammation and produce mucus; the main component of respiratory mucus is mucin (MUC), which has antiviral and anti-inflammatory functions. However, excessive mucin production can lead to obstruction of the airway lumen, restriction of airflow, and ventilation dysfunction. In addition, excess mucus can reduce mucociliary clearance and the local defense of the respiratory tract, allowing foreign bodies and pathogenic bacteria to remain in the lungs and airways, and causing destructive pathological processes to the body’s inherent immune clearance system [7]. MUC5AC is the most important mucin in airway mucus studies in disease states. Studies have shown that tight junctions (TJs) of airway epithelial cells are involved in regulating the mechanisms of signal transduction of epithelial cell proliferation, gene expression, differentiation, and morphogenesis [8]. Claudins are TJ membrane proteins that are expressed in epithelia and have been reported to be directly associated with epithelial differentiation and to promote excessive mucus secretion [9]. Recent reports suggest that EGFR down-regulates the expression of claudin1 in bronchial epithelial cells after activation by its ligand, and the downregulation of claudin1 promotes the expression of MUC5AC and exacerbates airway inflammation [10].

Our previous study found that the expression of MUC5AC and amino-terminal kinase (JNK1) in airway of PM2.5-exposed rats was adjusted upwards with the increase of PM2.5 exposure concentration, and the apoptosis rate of cells also increased significantly [6]. Huang et al. [11] reported that wood fumes (WSPM2.5) induced the production of MUC5AC in primary human airway epithelial cells and NCI-H292 cell lines, an induction process mediated by activation of epithelial growth factor receptor (EGFR)/extracellular signal-modulating kinase (ERK) signaling, which is mediated by the EGFR ligand-dependent mechanism. Therefore, PM2.5 induces the activation of the EGFR/MAPK signaling pathway, and the regulatory mechanism of high expression of MUC5AC, excessive secretion of airway mucus, and aggravation of airway inflammation through the downregulation of claudin1 is worth studying.

MiRNAs are small non-coding RNAs capable of inducing multidirectional regulation of gene expression and a wide range of biological processes. MiRNAs have been shown to be extensively involved in physiological and pathological processes of tissue damage and repair. Recent studies have found high expression levels in both arms (-3p and -5p) of miR-133a in group B of patients with chronic obstructive pulmonary disease (COPD) [12, 13]. The expression of miR-133a-3p and -5p in circulating extracellular vesicles (EV) is associated with their expression in plasma and exhaled respiratory condensate in patients with COPD [14]. We found that miR-133b-5p can target binding to EGFR through bioinformatics database predictive analysis, and found that the regulatory imbalance of miRNA is closely related to atmospheric particulate matter and motor vehicle exhaust particles *in vivo* and *in vitro* experiments in animals [15–17].

Based on the aforementioned findings, we hypothesized that PM2.5 exposure inhibits the expression of miR-133b-5p, activates the EGFR/MAPK signaling pathway through down-regulation of miR-133b-5p, downregulates the expression of claudin1 in bronchial epithelial cells, and promotes the production of MUC5AC, leading to increased secretion of airway mucus, which consequently exacerbates airway inflammation. However, there are currently no reports at home and abroad on the effect and mechanism of miR-133b-5p on PM2.5-related airway mucus in rats. The aim of this study was to investigate the mechanism by which miR-133b-5p regulates PM2.5-related airway mucus hypersecretion, and to provide a new ideas for the treatment of PM2.5-related airway mucus hypersecretion and airway inflammation from a genetic perspective.

## RESULTS

### Expression of miR-133b-5p in rat lung tissue

**Figure 1:**
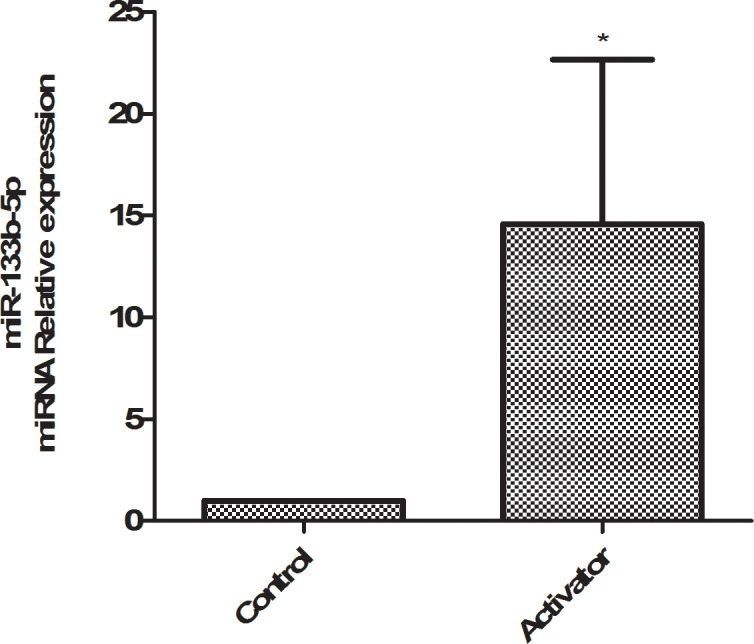
qPCR detects the expression of miR-133b-5p in rat lung tissue.

A tail vein was injected with miR-133b-5p activator (miR-133b-5p agomir, 10nmol, *in situ* injection of lung tissue two days before death), qPCR was used to detect the expression of miR-133b-5p in rat lung tissue, and the results showed that the expression of miR-133b-5p in rat lung tissue in the miR-133b-5p activator group was significantly higher than that in the blank control group, as shown in Figure 1.

### Validation of miR-133b-5p targeting binding target gene EGFR

**Figure 2:**
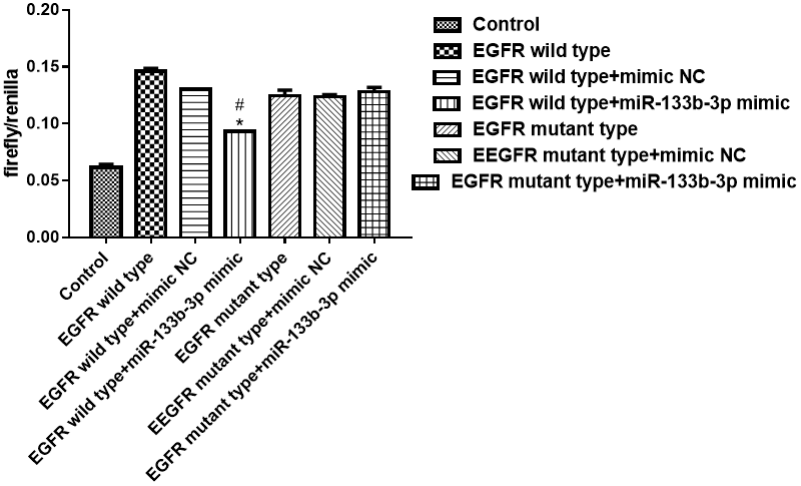
Dual-luciferase validated miR-133b-5p targeted binding target gene EGFR analysis.

By constructing the dual-luciferase reporter vector, blank reagent group, the target gene EGFR wild-type dual-luciferase reporter vector group, the target gene EGFR wild-type dual-luciferase reporter vector + mimic NC group, the target gene EGFR wild-type dual-luciferase reporter vector + miRNA mimic group, the target gene EGFR mutant dual-luciferase reporter vector group, the target gene EGFR mutant dual-luciferase reporter vector + mimic NC group, the target gene EGFR mutant dual-luciferase reporter vector +miRNA mimic group was grouped with transfection of 293T cells, and the fluorescence spectrophotometer detected the fluorescence value, and the results showed that miR-133b-5p targeted binding to the target gene EGFR, the results were shown in Figure 2.

### Rats PM2.5 infection model HE model validation

**Figure 3:**
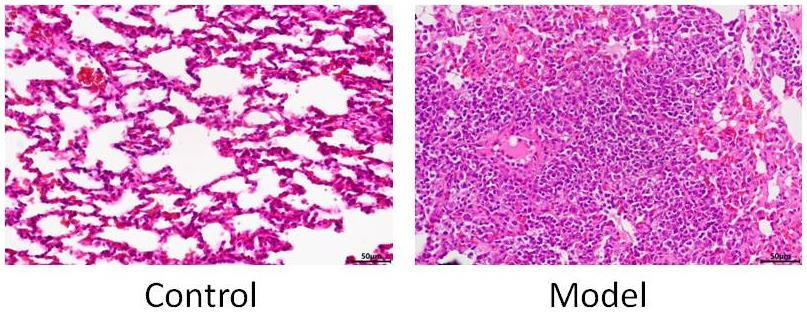
HE staining model validation.

Compared with the control group, the alveolar structure of the model group was destroyed, focal inflammatory cells appeared in the lung stroma and lung parenchyma, a large amount of mucus was visible in the lung interstitium, and the rat PM2.5 exposure model was successful, as shown in Figure 3.

### ELISA measures inflammatory indicators in the serum of each group

**Figure 4:**
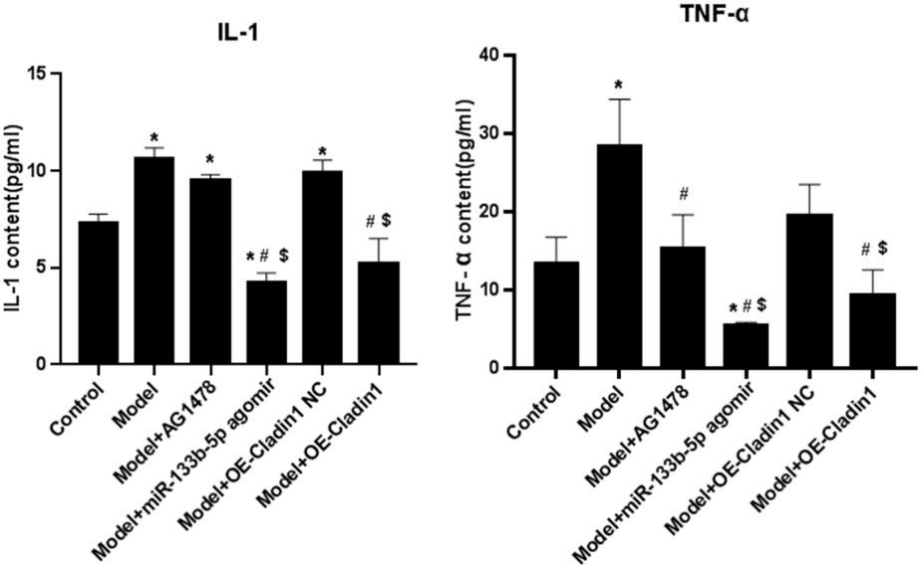
The content of inflammatory indicators IL-1 and TNF-α in the serum of rats in each group (*P<0.05, compared with the control group; ^#^*P*<0.05, compared with the model group; ^$^*P*<0.05, compared with the model + Claudin1 overexpression no-load group).

The content of IL-1 and TNF-α in the serum of rats in each group was detected by ELISA, and the results showed that the expression of IL-1 and TNF-α in the model group was significantly higher than that of the control, and the expression of IL-1 and TNF- in the model group was significantly higher than that in the model group, and in the model + AG1478 treatment group compared with the model group, and in the model + miR-133b-5p agomir treatment group compared with the control group, the model group and the model + Claudin1 overexpression no-load group, and in the model + The Claudin1 overexpression group is down-tuned compared to the model group and the model + Claudin1 overexpression no-load group, as shown in Figure 4.

### HE staining to observe the pathology of lung tissue

**Figure 5:**
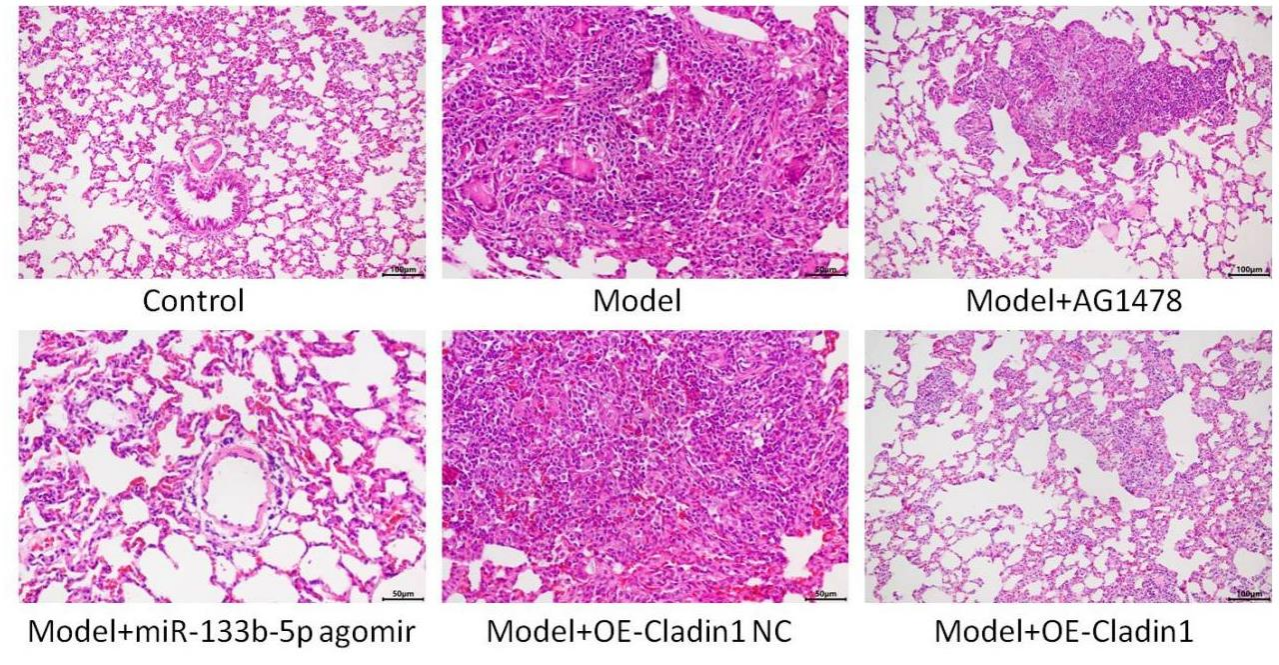
HE staining to observe the lung tissue of rats in each group.

HE staining observation model group and model + Claudin1 overexpression blank load group of lung tissue has obvious interstitial edema, capillary wall expansion, accompanied by a large number of monocytes, lymphocytes and neutrophils large infiltration, alveolar cavity visible significant bleeding; In the lung tissues of the model + AG1478 treatment group, the model + miR-133b-5p agomir treatment group, and the model + Claudin1 overexpression group, the capillary dilation and congestion decreased, the degree of stromal edema decreased, and the infiltration of lymphocytes, monocytes, and neutrophils decreased, as shown in Figure 5.

### WB detects the expression of lung histone proteins

**Figure 6:**
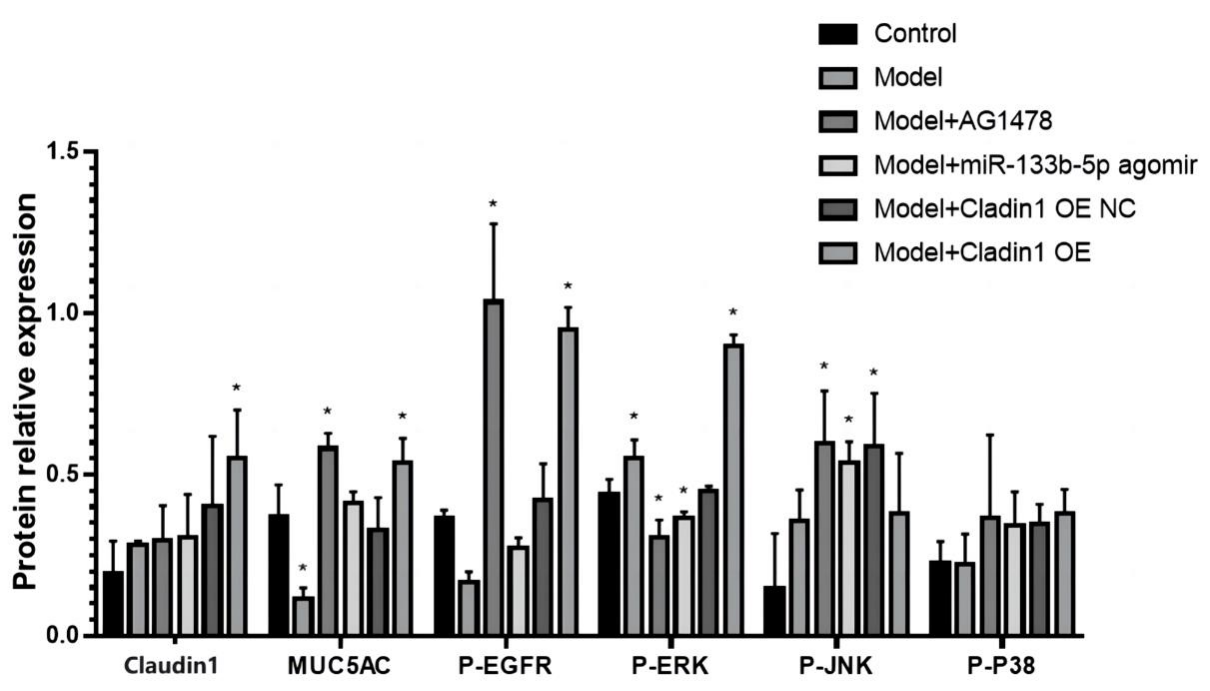
WB detects the expression of proteins associated with lung tissue.

The expression of p-EGFR, MUC5AC, p-ERK1/2, p-JNK, p-P38, Claudin1 protein was detected by WB, and the results showed that Claudin1 had the highest expression in the model + Claudin1 overexpression group, the MUC5AC and p-EGFR proteins were down-regulated in the model group, and the expression was upregulated in the model +AG1478 treatment group and the model + Claudin1 overexpression group, p-ERK1/2, The expression of p-JNK was upregulated in the model group, and there was no significant difference in the expression of the p-P38 protein in each group (Figure 6).

### Immunohistochemistry detects the expression of relevant proteins

**Figure 7:**
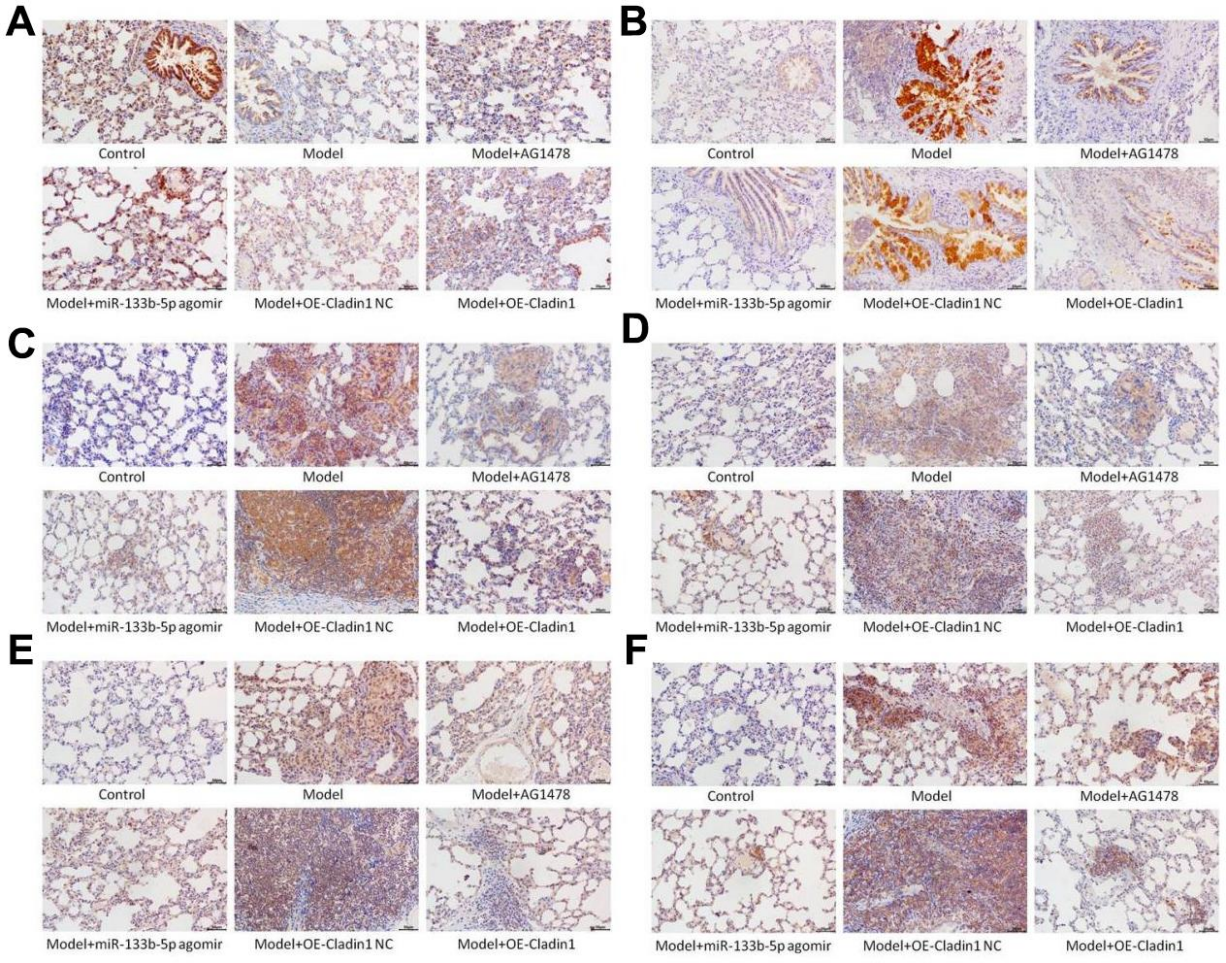
**Immunohistochemistry detects the expression of proteins in lung tissue note:** (**A**) Claudin1 protein expression; (**B**) Expression of MUC5AC protein; (**C**) p-EGFR protein expression; (**D**) p-ERK1/2 protein expression; (**E**) p-JNK protein expression; (**F**) P-P38 protein expression.

The expression of p-EGFR, MUC5AC, p-ERK1/2, p-JNK, p-p38 and Claudin1 proteins in lung tissues was detected by immunohistochemistry, and the results showed that the expression of p-EGFR, MUC5AC, p-ERK1/2, p-JNK, p-p-p38 protein was increased and Claudin1 protein expression decreased in the model group compared with the control group. There was no significant difference in the expression of the six proteins in the model group and the model + Claudin1 overexpression blank load group. In the model + AG1478 treatment group, the model + miR-133b-5p agomir treatment group and the model + Claudin1 overexpression group, compared with the model group, p-EGFR, MUC5AC, p-ERK1/2, p-JNK, p-p38 protein expression down-regulated, Claudin1 protein expression upregulated, the results are shown in Figure 7.

## DISCUSSION

In recent years, the impact of air pollution on human health has been the focus of public attention. Epidemiological studies have shown an increasing incidence and case fatality rate of respiratory diseases such as COPD, bronchial asthma, and lung cancer as PM2.5 concentrations in the air increase [18, 19]. PM2.5 has been reported to be associated with a variety of respiratory diseases, [20] but the specific mechanism is unclear. The results of the current study showed that after the rats were exposed to PM2.5, the alveolar structure was destroyed, the capillary wall was expanded, and accompanied by a large number of monocytes, lymphocytes and neutrophils were infiltrated in large quantities, and a large amount of mucus was visible in the interstitium of lungs. IL-1, TNF-α are inflammatory cytokines, associated with airway inflammation and systemic inflammation, ELISA elicited that IL-1, TNF-α in the serum of in rats increased after the exposure to PM2.5. Consistently, airway inflammation in rats exposed to PM2.5 was aggravated.

While the expression of certain miRNAs may be affected by PM2.5 exposure [21], our findings suggest that the expression of miR-133b-5p is downregulated after rat are exposed to PM2.5. To further validate the role of miR-133b-5p, miR-133b-5p agomir overexpressed miR-133b-5p. The results showed that the serum IL-1 and TNF-α were significantly down-regulated in the PM2.5 infection model + miR-133b-5p agomir treatment group compared with the control group, the model group and the model +Claudin1 overexpression blank load group, and were down-regulated in the model +Claudin1 overexpression group compared with the model group and the model +Claudin1 overexpression blank load group, and in the model +AG1478 treatment group. At the same time, in the lung tissues of the model +miR-133b-5p agomir treatment group, the model + AG1478 treatment group and the model + Claudin1 overexpression group, the capillary expansion, congestion decreased, and the degree of interstitial edema decreased, and the infiltration of lymphocytes, monocytes and neutrophils decreased, and it was verified that miR-133b-5p and Claudin1 overexpression inhibited PM2.5-induced airway inflammation and alleviated lung damage, which suggests that PM2.5 exposure can lead to greater lung damage and histopathological changes, and ultimately promote the development and progression of airway inflammation [22].

Our previous study found that with the increase of PM2.5 exposed dose, the expression of MUC5AC and JNK1 at the protein and mRNA levels in airway epithelial cells of experimental rats increased [6]. Related studies have also shown that EGFR downregulates the expression of claudin1 in bronchial epithelial cells after activation by its ligand, and the downregulation of claudin1 promotes the expression of MUC5AC and exacerbates airway inflammation [10]. Through bioinformatics database predictive analysis, we found that miR-133b-5p can target binding EGFR, and dual-luciferase results verify that miR-133b-5p can target binding target gene EGFR. We speculate that miR-133b-5p has an effect on PM2.5-related airway inflammation and hypersecretion of airway mucus through the EGFR/MAPK pathway. The protein detection results show that compared with the control group, the expression of p-EGFR, MUC5AC, p-ERK1/2, p-JNK and p-p38 proteins was increased and Claudin1 protein expression decreased in the PM2.5 exposed model group. There was no significant difference in the expression of the six proteins in the model group and the model + Claudin1 overexpression blank load group. In the model + AG1478 treatment group, model + miR-133b-5p agomir treatment group and model + Claudin1 overexpression group, compared with the model group, p-EGFR, MUC5AC, p-ERK1/2, p-JNK, p-p38 protein expression was down-regulated, and Claudin1 protein expression was up-regulated. The results showed that the expression levels of EGFR and MUC5AC were down-regulated by miR-133b-5p, and the expression levels of Claudin1 protein were up-regulated, while the overexpression of Claudin1 protein could inhibit the expression of MUC5AC. The expression of miR-133b-5p were down-regulated in rats after PM2.5 exposure, which activated EGFR in rat lung tissue and promoted the expression of MUC5AC by downregulating the expression of claudin1 in bronchial epithelial cells. Huang et al. [11] reported that wood fumes (WSPM2.5) induce the production of MUC5AC in primary human airway epithelial cells and NCI-H292 cell lines, an induction process mediated by the activation of epithelial growth factor receptor (EGFR)/extracellular signal-modulating kinase (ERK) signaling, mediated by the EGFR ligand-dependent mechanism. This is consistent with the upregulation of the expression of EGFR and MUC5AC in PM2.5 exposed rats in our study. The overexpression of Claudin1 protein inhibits the secretion of MUC5AC, weakens the airway inflammation and excessive secretion of airway mucus induced by PM2.5 exposure. In general, miR-133b-5p targeted EGFR, PM2.5 exposure inhibited the expression of miR-133b-5p, and the down-regulated miR-133b-5p inhibited Claudin1 protein expression by inducing the activation of the EGFR/MAPK signaling pathway, thereby activated and promoted MUC5AC production, caused the continuous secretion of airway mucus, aggravated airway inflammation, leaded to the progression of various chronic airway inflammatory diseases.

## CONCLUSIONS

In summary, our study found a new mechanism of PM2.5 associated with hypersecretion of airway mucus and airway inflammation. Exposure to PM 2.5 inhibited the expression of miR-133b-5p in rats, activated the EGFR/MAPK pathway, and caused excessive secretion of MUC5AC and aggravated airway inflammation by inhibiting the expression of Claudin1 protein. If claudin1 can be restored by inhibiting the EGFR cascade, which could reduce secretion of airway MUC5AC and control airway inflammation. This would be a novel and effective treatment strategy for the treatment of PM2.5-related chronic airway inflammatory diseases, but the findings should be interpreted as exploratory given the rat model limitations and early stage of testing. Moreover, further research is needed on whether miR-133b-5p can cause PM2.5-associated hypersecretion of airway mucus and airway inflammation through other mRNAs and pathways, and whether upstream lncRNAs are used to regulate miR-133b-5p, and the EGFR/MAPK pathway still remains to be explored.

## MATERIALS AND METHODS

### Preparation of PM2.5

PM2.5 was sampled in the tenth floor of the outpatient building in Jiangxi Provincial Chest Hospital (about 30 meters high) by TSP / PM10 / PM2.5 Particle Sampler (Beijing Geological Instrument and Dick Mechanical and Electrical Technology Co., Ltd., Beijing, China). Sampling points around were densely populated without industrial enterprises. A glass fiber filter membrane (90 mm) was applied to collect PM2.5 samples in a 24h continuous model from March to June 2020. The sampling was stopped for rain, snow, etc. The filter membrane containing PM2.5 was then immersed in distilled water and oscillated by an ultrasonic oscillator. The sample was filtrated by multi-layer sterile gauze. The filtrate was centrifuged at 4° C (10000 r/min) for 20 min, and the supernatant was collected. The supernatant was oscillated by ultrasonic oscillator and the particulate matter at the bottom was collected. Finally, the supernatant and the collected particles were mixed and underwent vacuum freeze drying, and the PM2.5 suspension was diluted with sterile water into 40μg/mL and was stored at 4° C for later use.

### Experimental reagents and equipment

Rats Tumor Necrosis Factor (TNF-α) Kit (MM-0180R1, Enzyme-Free), Rats Interleukin 1 (IL-1) Kit (MM-0046R1, Enzyme-Free), Trizon Reagent (CW0580S, CWBIO), miRNA Extraction Kit (CW0627S, CWBIO), miRNA 1st Strand cDNA Synthesis Kit (by stem-loop) (MR101-02, Vazyme), miRNA Universal SYBR qPCR Master Mix(MQ101-02, Vazyme), hematoxylin staining solution (ZLI-9610, Zhongyi Jinqiao), Yihong staining solution (G1100, Solarbio), ultra-clean advanced film gel (YZB, BASO), Scott blue-blue solution (G1865, Solarbio), P-EGFR (AF30314, Affinity), Claudin1 (AF0127, Affinity), MUC5AC(A17325, Abclonal), P-ERK1/2(AF1015, Affinity), P-JUK(AF3318, Affinity), P-P38(AF4001, Affinity); Horseradish labeled goat anti-rabbit IgG (H+L) (ZB-2301, Nakasugi Jinqiao), DAB color rendering kit (CW0125, CWBIO), neutral resin (CW0136, CWBIO), complete DMEM (high sugar) medium (KGM12800S, Keygen Bio), incomplete DMEM (high sugar) medium (KGM12800N, Keygen Bio), 293T (BNCC100530, North Na Biologics), mimic NC (courtesy of Zhonghong Molecular Laboratory), miR-133b-5P mimic (provided by Zhonghong Molecular Laboratory), Lipofectamine 3000 Transfection Reagent (L3000015, Invitrogen), OPTI-MEM medium (31985-062, Gibco), Biluciferase Reporter Detection Kit (RG027, Biyuntian), Sea kidney plasmid pRL-SV40 (provided by Zhonghong Molecular Laboratory), EGFR wild-type luciferase vector (provided by Zhonghong Molecular Laboratory), EGFR mutant luciferase vector (provided by Zhonghong Molecular Laboratory). Pipette (100-1000ul, Eppendorf), pipette (20-200ul, Eppendorf), pipette (5-50ul, Eppendorf), automatic microplate reader (WD-2102B, June 1st), refrigerated freezer (BD/BC-415DKEM, Midea), electric thermostatic incubator (DHP-9054, Shandong Brocade Bioindustry Co., Ltd.) -80° C refrigerator (BDF-86V348, BIOBASE), medical cryopreservation box (DW-25L262, Haier), automatic sample rapid grinder (Tiss-12, Shanghai Jingxin Industrial Development Co., Ltd.), high-speed desktop refrigerated centrifuge (H1750R, Hunan Xiangyi Laboratory Instrument Development Co., Ltd.), microcentrifuge (D1008E, SCJLOGEX), vortex mixer (XH-C, Changzhou Yuexin Instrument Manufacturing Co., Ltd.), ultraviolet spectrophotometer (NP80, NanoPhotometer), ordinary PCR amplification instrument (TC-EA, Hangzhou Bori Technology Co., Ltd.), fluorescence cycler (CFX Connect™ real-time, Bó Lè Life Medical Products (Shanghai) Co., Ltd.), pharmaceutical refrigerator (BYC-310, Shandong Brocade Biologics);, microscope (BX43, OLYMPUS), microtome (2235, Leica); Electric blower drying box (HGZF-101-1, Shanghai Yuejin Medical Device Co., Ltd.), pharmaceutical refrigeration cabinet (BYC-310, Shandong Brocade Biologics); Electric thermostatic blower drying chamber (HGZF-101-1, Shanghai Yuejin Medical Device Co., Ltd.), thermostatic incubator (DHP-9054, Shandong Brocade Biologics), microscope (BX43, OLYMPUS), pressure cooker (YS20ED, Suber); Induciton cooker (HK-22, Hanke Electric Factory, Dongfeng Town, Zhongshan City), CO2 Incubator (BPN-80CW, Shanghai Yiheng Scientific Instrument Co., Ltd.), Multifunctional Microplate Analyzer (SuPerMax 3100, Shanghai Flash Spectrum Biotechnology Co., Ltd.)

### Laboratory animals and grouping

Sixty specific pathogen-free (SPF) male Wistar rats, aged 6-8 weeks, with weight of 220±20g, were purchased from Pengyue Experimental Animal Breeding Co., Ltd, Jinan, Shandong, China (license no. SCXK[Lu]20190003). The rats were randomly divided into the following six groups: 1) Blank control group: normal saline; 2) Model group: PM2.5 suspension; 3) Model + AG1478 treatment group: after PM2.5 suspension treatment, subcutaneously inject EGFR-specific blocker (AG1478, 17.5 mg/kg, twice a week); 4) Model +miR-133b-5p agomir treatment group: after PM2.5 suspension treatment, the lung was injected with miR-133b-5p activator *in situ* (miR-133b-5p agomir, 10nmol, injected 48h before death); 5) Model + Claudin1 overexpression no-load group: after PM2.5 suspension treatment, tracheal instillation Claudin1 overexpression adenovirus no-load; 6) Model + Claudin1 overexpression group. After PM2.5 suspension treatment, tracheal instillation of Claudin1 overexpression adenovirus vector. Ten rats in each group. PM2.5 suspension (40μg/mL) was atomized by an ultrasonic nebulizer to administered to the above experimental groups except the control group. The PM2.5 suspension was administered once a day for 1 hour each time for 4 weeks.

### Reverse transcription quantitative polymerase chain reaction (RT-qPCR)

The lung tissue sample miRNA was extracted by Trizon lysate, the purity and concentration of miRNA were analyzed by ultraviolet spectrophotometer, the miRNA 1st Strand cDNA Synthesis Kit was reversed transcription, and the expression of miR-133b-5p was calculated by using cDNA as the template and detected on the PCR tester, and U6 or β-actin was used as the internal reference to calculate the expression of miR-133b-5p in each group of samples. The primer sequences are shown in Table 1. Operating System is shown in Table 2. A three-step method was used in the laboratory, and the specific reaction procedure is shown in the following Table 3.

**Table 1:** The primer sequences.

**Primer name**	**Primer sequence (5’-3’)**	**Product length (bp)**	**Annealing temperature (° C)**
miR-133b-5p F	GCGGCTGGTCAAACGGAA	65	58
miR-133b-5p R	AGTGCAGGGTCCGAGGTATT		
miR-133b-5p RT	GTCGTATCCAGTGCAGGGTCCGAGGTATTCGCACTGGATACGACACTTGG	50	
U6 F	ATTGGAACGATACAGAGAAGATT	90	58
U6 R	GGAACGCTTCACGAATTTG		

**Table 2:** Operating system.

**regent (RNA)**	**Volume (20ul Reaction system)**
2 × ChamQ Universal SYBR qPCR Master Mix	10ul
cDNA	1ul
Upstream primers	0.4ul
Downstream primers	0.4ul
RNase Free ddH_2_0	8.2ul

**Table 3:** Three-step method of laboratory.

**Steps**	**Temperature**	**Time**	**The number of cycles**
Prevariation	95° C	10min	1
denaturation	95° C	10s	40
anneal	58° C	30s	
extend	72° C	30s	

### Western blot (WB)

Tissue samples were removed with forceps and 50 mg of samples were put into centrifuge tubes with lysate added, and then grinded in a grinder and centrifuged at 12000 r/minute for 10 minutes. The supernatant was taken (BCA assay) and added with buffer solution and then boiled for five minutes and stored at -20° C. Protein concentrations were detected with the BCA egg quantification kit. Configure 10% SDS-PAGE Separation Gel and 5% Concentrated Gel for glue, then add protein samples and Marker and perform electrophoresis, cut the glue and put away the “sandwich” (sponge-filter paper-glue-membrane-filter paper-sponge). 300mA constant flow membrane for 90 minutes. 3% skim milk was closed at room temperature for one hour. Incubate the PVDF membrane for primary antibody overnight. Incubated the secondary antibody with the PVDF membrane for two hours after washing the membrane. After washing the film, soaked the PVDF film with luminescence solution, and then placed it in the sample placement area of the ultra-high sensitivity chemiluminescence imaging system to run the program development imaging.

### Enzyme-linked immunosorbent assay (ELISA)

1) After 60 minutes of equilibration at room temperature, the required lath was removed from the aluminum foil bag, and the remaining lath was sealed back to 4° C with a self-sealing bag; 2) The standard wells and sample wells were set, and 50μl of standard materials of different concentrations were added to the standard wells; 3) 50μl of the sample were added to be tested to the sample well; Blank holes were not added; 4) 50μl of biotin-labeled antibody were added to each well, sealed the reaction well with sealing film, and incubated in a 37° C incubator for 30 minutes; Standard hole blank hole was not added; 5) Discard the liquid, patted dry on the absorbent paper, filled each well with washing liquid (350μl), let stand for 1min, shaked off the washing liquid, patted dry on the absorbent paper, and repeated washing the plate five times; 6) In addition to the blank wells, 100μl of horseradish peroxidase (HRP)-labeled detection antibody was added to each well of the standard wells and sample wells, and the reaction wells were sealed with a sealing film, 37° C incubator incubation 30 minutes; 7) Discarded the liquid, patted dry on the absorbent paper, filled each well with washing liquid (350μL), let stand for 1min, shaked off the washing liquid, patted dry on the absorbent paper, and repeat washing the plate five times; 8) 50μl of substrate A and B were added to each well and incubate at 37° C away from light for 15 minutes; 9) 50μl of stop solution were added to each well, within 15 minutes, and determined the OD value of each well at a wavelength of 450 nm.

### HE staining

The tissue was removed and rinsed with running water for several hours, and then dehydrated by 70%, 80%, 90% ethanol solution at all levels, pure alcohol, xylene mixture of 15 minutes, xylene I.15 minutes, II.15 minutes (until transparent). Half of the mixture of xylene and paraffin was placed in for 15 minutes, and then paraffin I and paraffin II were placed for 50-60 minutes each. Paraffin embedding, slicing. The paraffin slices were baked and then dewaxed and hydrated. The slices that had been distilled into distilled water were placed in hematoxylin aqueous solution for three minutes, ethanol hydrochloride differentiation solution differentiation for 15 seconds, slightly washed, blue return to blue liquid for 15 seconds, rinsed by running water, red staining for three minutes, rinsed with water, dehydrated, transparent, sealed, microscopic inspection.

### Alcian blue periodic acid Schiff (AB-PAS) staining

Mucins in mucus contain mucopolysaccharides, so PAS staining can be used to show mucins. The staining process was as follows: The sections was routine dewaxed to water and rinsed with distilled water for 2 minutes. Then it was stained with Alcian blue staining solution for 13 minutes and rinsed with distilled water for 2 minutes for three times. Periodic acid solution dropwise and oxidized for 5 minutes, and rinsed with distilled water for 2 minutes. Then dyed with Schiff Reagent solution for 13 minutes and rinsed with running water for 10 minutes. Last, sections were dehydrated with ethanol, transparent by xylene and sealed with neutral gum like HE staining. The airway epithelium of each group was observed under the light microscope, and the mucinous material was stained purplish red after PAS staining, and the classically rounded proximal airways were selected for image acquisition. The images were analyzed with Image-Pro Plus 6.0 for assessment of quantification of mucus.

### Immunohistochemical detection of proteins

(1) Paraffin slicing pretreatment: 1) Baking slices: Slices of tissue were put into a 65° C oven for two hours; 2) Dewaxing: Slice in xylene for 10 minutes, replaced xylene for another 10 minutes; 3) Hydration: Slices were put into 100% ethanol, 100% ethanol, 95% ethanol, 80% ethanol and purified water for 5 minutes each. (2) Antigen repair: Put the slices into the repair box, added the antigen repair solution (citric acid buffer), the pressure cooker was heated until it automatically deflates, left the heat source after two minutes to cool naturally, discarded the antigen repair solution, and rinsed the slice with PBS. (3) Elimination of endogenous peroxidase: The sections were moved into a wet box, freshly prepared 3% hydrogen peroxide was added to remove endogenous peroxidase blocking solution, incubated at room temperature for 10 minutes, PBS eluted fully. (4) Blocking: Non-specific sealing: PBS was immersed slides for three times, each time for five minutes, absorbent paper blotted PBS around the tissue, added 5% BSA dropwise on the slide, block at 37° C for 30 minutes. (5) Immune response: 1) Apply primary antibody: aspirated the blocking liquid around the tissue with absorbent paper, did not wash, added a sufficient amount of diluted primary antibody to each slide: RIPK3 (1:150), put it in a wet box, incubated at 4° C overnight. 2) Apply the secondary antibody: removed 4° C and incubated the overnight wet box, let stand at room temperature for 45 minutes, PBS was immersed slides three times, five minutes each time, added horseradish labeled goat anti-rabbit IgG (H+L) (1:150), incubated at 37° C for 30 min, PBS eluted fully. (6) Chromogenic counterstaining: 1) Developed DAB color for 5-10 minutes, mastered the degree of staining under the microscope, PBS or tap water rinsed for one minutes; 2) Hematoxylin counterstained for three minutes, alcohol hydrochloride differentiation, returned to blue; 3) Tap water rinsed for one minutes, dehydrate, transparent, seal, microscopy.

### Luciferase assay

Platening: Laid 12-well plates on the cells according to the needs of the experiment, digest, count, dilute according to the needs of the experiment, 8×104 cells per well, evenly spread into the cell culture plate, make a label, placed in the incubator for culture, and carried out transfection experiments after the cells were fully adhered. Transfection: When the cell density reached 70%, prepared for transfection, changed the cells before transfection, and changed to low-serum medium (5% FBS). Hands-on detection: each group took 70ul of cell lysate, added it to the black 96-well plate, added 100ul of luciferase detection reagent per well, and detected the activity of firefly luciferase on the machine; Then added the sea kidney luciferase detection reagent (detection substrate: buffer = 1:100) to each well,100ul per well, and detected the sea kidney luciferase activity on the machine.

### Statistical analysis

SPSS20.0 statistical software was used to analyze data. All experiments were repeated three times and the quantitative results were expressed as mean ± standard deviation. The quantitative numerical comparison between the two groups was carried out by independent sample *t* test, the quantitative numerical comparison between multiple groups was analyzed by one-way ANOVA, and the *S-N-K* method was used for two-two comparison. Inspection level α = 0.05.
